# Global remapping emerges as the mechanism for renewal of context-dependent behavior in a reinforcement learning model

**DOI:** 10.3389/fncom.2024.1462110

**Published:** 2025-01-15

**Authors:** David Kappel, Sen Cheng

**Affiliations:** Institute for Neural Computation, Faculty of Computer Science, Ruhr University Bochum, Bochum, Germany

**Keywords:** hippocampus, global remapping, reinforcement learning, extinction learning, place cell

## Abstract

**Introduction:**

The hippocampal formation exhibits complex and context-dependent activity patterns and dynamics, e.g., place cell activity during spatial navigation in rodents or remapping of place fields when the animal switches between contexts. Furthermore, rodents show context-dependent renewal of extinguished behavior. However, the link between context-dependent neural codes and context-dependent renewal is not fully understood.

**Methods:**

We use a deep neural network-based reinforcement learning agent to study the learning dynamics that occur during spatial learning and context switching in a simulated ABA extinction and renewal paradigm in a 3D virtual environment.

**Results:**

Despite its simplicity, the network exhibits a number of features typically found in the CA1 and CA3 regions of the hippocampus. A significant proportion of neurons in deeper layers of the network are tuned to a specific spatial position of the agent in the environment—similar to place cells in the hippocampus. These complex spatial representations and dynamics occur spontaneously in the hidden layer of a deep network during learning. These spatial representations exhibit global remapping when the agent is exposed to a new context. The spatial maps are restored when the agent returns to the previous context, accompanied by renewal of the conditioned behavior. Remapping is facilitated by memory replay of experiences during training.

**Discussion:**

Our results show that integrated codes that jointly represent spatial and task-relevant contextual variables are the mechanism underlying renewal in a simulated DQN agent.

## 1 Introduction

Classical Pavlovian conditioning has taught us that animals can learn to emit a conditioned response (CR) to a conditioned stimulus (CS) when the CS is repeatedly paired with a physiologically relevant, unconditioned stimulus (US), which usually involves reinforcement or punishment (Pavlov, 1927). When the CS is subsequently presented repeatedly without the US, the association between CR and CS weakens, a phenomenon referred to as extinction learning (Auchter et al., 2017). However, this effect has been found to be complex and highly context-dependent, which can lead to renewal of learned behavior under certain circumstances (Dunsmoor et al., 2015).

This effect is most evident in the ABA renewal paradigm (Bouton, 2004; Corcoran, 2004; Ji and Maren, 2008; Fujiwara et al., 2012; Zelikowsky et al., 2013). In this experimental design, a subject typically acquires a CR to a CS when it is paired with an US in a particular context A, e.g., defined by a spatial enclosure, an odor or light stimulus, and then the response is extinguished in a different context B in the absence of the US. When the subject returns to context A (without the US), the CR is restored. The ABA renewal phenomenon suggests that extinction learning does not completely erase or overwrite the previously learned association, but rather forms a new contextual association (Dunsmoor et al., 2015).

**Figure 1:**
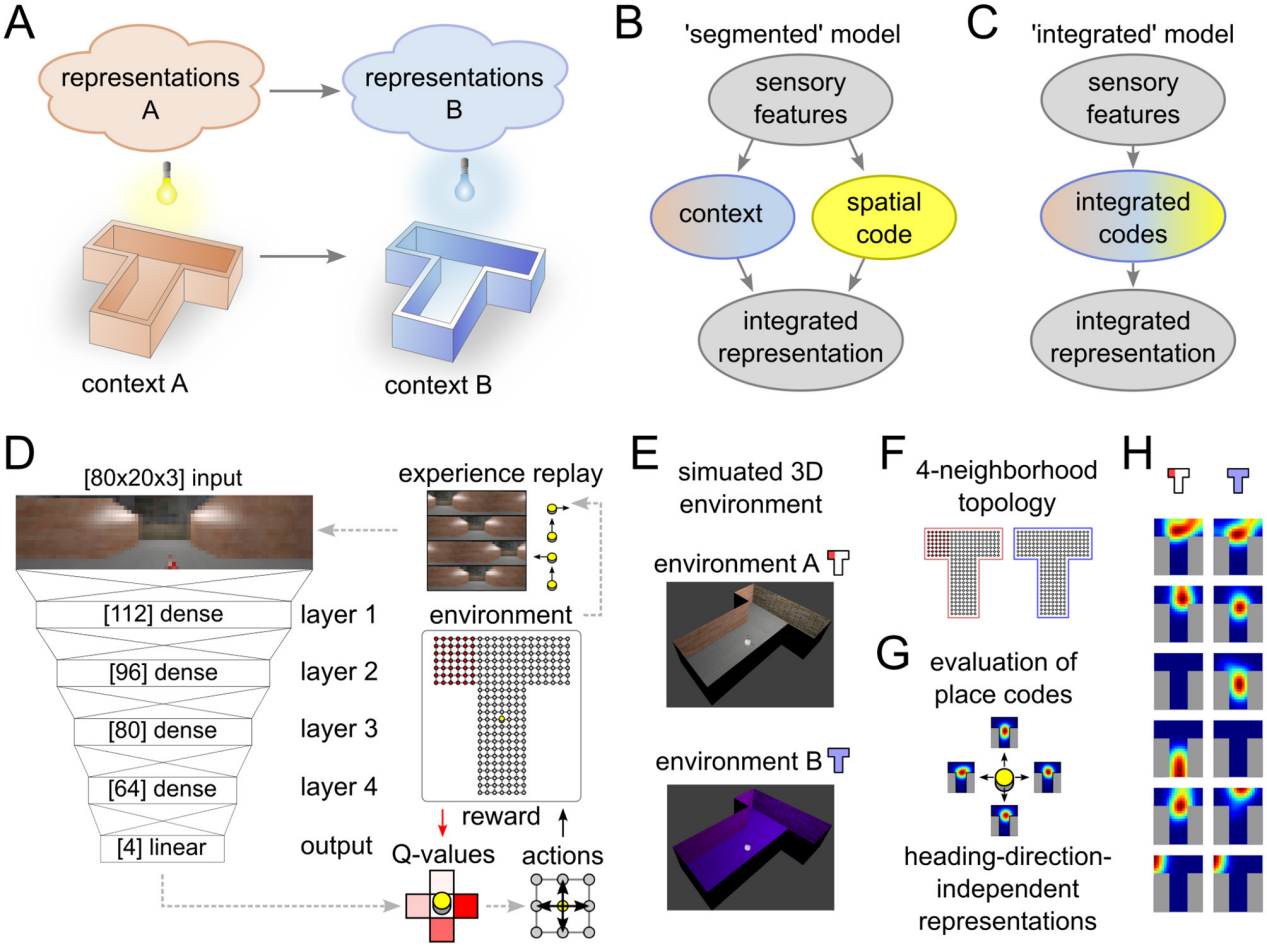
Experimental setup and data analysis. **(A)** Illustration of remapping paradigm. Moving from environment A to B results in the expression of a different spatial representation. **(B, C)** Two alternative models that can explain place field remapping, the “segmented” model **(B)** separates codes for space and context, while the “integrated” model **(C)** combines these codes. **(D, E)** Illustration of the reinforcement learning agent **(D)** that interacts with a simulated environment **(E)**. **(F–H)** Place field analysis performed on the neural activations. 4-neighborhood topology in the T-maze **(F)** is used to record neural activity maps for each heading direction **(G)**. Activity of representative example neurons that showed place-cell-like behavior in one or both environments **(H)**.

The hippocampal formation plays an important role in extinction learning (Jones et al., 2016; Bernier et al., 2017; Hainmueller and Bartos, 2020), and it was found that extinction learning depends on learning mechanisms in the hippocampus (Peters et al., 2010; Soliman et al., 2010; Rosas-Vidal et al., 2014; Wang et al., 2018; Bouton et al., 2021). Furthermore, the hippocampal formation shows context-dependent activity patterns and complex dynamics, e.g. place cell activity during spatial navigation in rodents, or place fields, remap between different environments (Grieves and Jeffery, 2017; Latuske et al., 2018) (Figure 1A). The latter phenomenon is referred to as global remapping and means that some place cells are active only on one environment, and not the other, and some place cells have place fields in different relative locations in the two environments (Leutgeb et al., 2005). Hippocampal place field maps also encode abstract task-relevant variables (Knudsen and Wallis, 2021). Some authors have therefore proposed that remapping is the physiological basis for task-relevant context variables (Kubie et al., 2020; Plitt and Giocomo, 2021; Sanders et al., 2020), i.e., that place field maps jointly encode place and context in an integrated code (Figure 1C).

Modern machine learning techniques, are now able to solve behavioral tasks that are comparable in complexity to real-world behavior and to reach human performance in some specific domains (Ashraf et al., 2021). These techniques have been used to model hippocampus functionality on an abstract level (Diekmann and Cheng, 2023) and to explain the emergence of complex task-relevant spatial codes (Vijayabaskaran and Cheng, 2022). Furthermore, in Walther et al. (2021), a simplified abstract ABA extinction learning paradigm was tested in a simulated agent. It was found that the prominent signs of extinction and renewal could be reproduced in the model, but the underlying representations of space and context that emerged in the circuit were not studied systematically.

A number of models for remapping have been proposed. An early model that focused on place cell remapping described the phenomenon as neural implementation of a planar path integrator for spatially tuned neurons (Redish et al., 1996; Zhang, 1996; McNaughton et al., 1996; Samsonovich and McNaughton, 1997), but assumed the existence of special signals to represent cues and contexts, and did not learn from realistic visual input. A related suggestion by Whittington et al. (2020) is that the remapping is the result of conjunctive coding in place cells that are driven by both invariant grid cells and non-invariant visual inputs when animals move between contexts. Other earlier models proposed to view the hippocampus as a hierarchical generative model (Stoianov et al., 2022; Taniguchi et al., 2022; Penny et al., 2013). Following these models, context can be recovered from network activity through statistical inference (Fuhs and Touretzky, 2007). A related idea also underlies an earlier statistical model of memory stability (Gershman et al., 2017). Based on these results, a general contextual inference theory of sensory-motor learning has recently been proposed (Heald et al., 2021). Redish et al. (2007) proposed a simplified computational model for extinction and renewal based on the explicit representation of contextual cues. Cochran and Cisler (2019) proposed an extension of the Rescorla-Wagner model (Rescorla, 1972) that relies on latent-state inferences to associate cues and rewards. This model was able to match a number of behavioral effects, such as spontaneous recovery, which previous models failed to reproduce. Another approach can disambiguate identical sensory inputs in two different contexts, if the sequence of observations differs between the contexts (George et al., 2021).

In contrast to these prior studies we focus here on the machanisms that enable remapping and renewal in a reinforcement learning setting. To this end, we develop a simulation environment that uses naturalistic inputs with implicit contextual information to study the emergence of representations in an ABA extinction and renewal paradigm. This allows us to study the emergent neural codes and behavior in a closed-loop simulation where an agent interacts with a 3-dimensional (3D) environment. Unlike previous models that focused mainly on symbolic representations of the environment and internal state of the agent, we directly use complex visual inputs to study ABA extinction and renewal. In our model, context-dependent spatial codes that closely resemble place cells, as well as a context-dependent remapping of these codes emerge from the learning rule and task demands.

The context-dependent codes represent task-relevant cues, as suggested by many previous models of extinction and renewal. In our model, these representations emerge automatically through learning in the complex environment. We compare two alternative hypotheses about the nature of contextual codes: A segmented code would suggest that separate populations of neurons encode context and spatial location (Figure 1B), whereas an integrated code would suggest mixed encoding of space and context (Figure 1C). We show the simultaneous emergence of spatial representations and task-relevant context variables throughout all layers of the reinforcement learning agent. These emerging representations in our model are more compatible with the integrated than with the segmented model. Furthermore, we show that the formation of independent spatial maps depends on the salience of the context stimulus. Finally, we investigate the role of experience replay in memory consolidation and stability of behavior and find a strong dependence between renewal and replay.

## 2 Methods

### 2.1 Simulated behavioral experiment

To test the behavior and spatial representations that emerge in the simulated agent during an ABA renewal paradigm, we designed a closed-loop simulation of a spatial task inspired by T-maze experiments (see Figures 1D, E). In every trial, the agent was located at the bottom center of the base of the T-maze and could move freely between grid points (238 in total) by choosing to move in one of four cardinal directions (North, East, South, West). Actions that would result in a movement outside the grid world consumed one time step, but otherwise had no effect. Heading directions of the agent were rotated in alignment of the actions in every time step. When a goal location (red) was visited, the agent received a reward (+20) and the trial was terminated. In every other time step, a negative reward of –1 was given to encourage the agent to find the goal as fast as possible. Unsuccessful trials were terminated after 400 time steps.

All simulations were based on the CoBeL-RL simulation framework (Diekmann et al., 2023). Visual feedback was provided to the artificial agent in the form of naturalistic images (Figures 1D, E) generated using the Blender 3D computer graphics software (version 2.79). The maze had a length of 2.8 m and walls were structured with photo-realistic textures. A 360° view image of a typical lab environment was projected onto a cylindrical sky box around the maze to provide distal spatial cues. A point light source with adjustable color was placed above the center of the T-maze. In the regular setting, the color of the light controlled the context (blue and white light condition). In the alternative setting, lighting conditions were not changed and context was established through an explicit signal. To generate the rendered input images, a camera object was placed at the agent's location and heading direction. Gaussian jitter with standard deviations 1 cm and 10° was added to the camera position and orientation, respectively. An image was then captured, scaled and cropped to 80 × 20 pixels, corresponding to an effective horizontal angle of view of around 240°.

### 2.2 Deep Q-learning network

To model spatial learning, we adopted reinforcement learning, and since the goal of this study was to analyze emergent spatial representations, we employed a Deep-Q Learning Network (DQN) agent. The DQN agent combines deep neural networks (DNNs) and Q-learning, i.e. the action-value function *Q*(*s, a*) is represented and approximated by a DNN (Ashraf et al., 2021). The update scheme of *Q* realizes one iteration of the Bellman equation, i.e. for the current state *s*, action *a*, reward *r*, and next state *s*′


$$Q\left(s,a\right)\leftarrow \left(1-\alpha \right)Q\left(s,a\right)+\alpha \left(r+\gamma \underset{a^{\prime }\in A}{\max}Q\left(s^{\prime },a^{\prime }\right)\right),$$

where α is a learning rate, γ is the reward discount factor and *A* is the state space of the actions. The discount factor γ depreciates future rewards and thus encourages fast goal seeking. Throughout all simulations, we used γ = 0.8.

### 2.3 Network architecture, experience replay and training procedure

A DNN was used to learn and infer *Q*-values *Q*(*s, a*) in response to complex visual stimuli. The DNN architecture in our agent consisted of a feed-forward densely-connected artificial neural network with four hidden layers (Figure 1D). Hidden layer sizes from input to output were 112, 96, 80, and 64. Batch normalization followed by rectified linear activation functions were used to create layer outputs. The inputs to the first layer of the DNN were 80 × 20 × 3 unprocessed RGB images. In the experimental condition with explicit context, a set of 160 neurons were added to the input to explicitly represent the experimental phase. 80 of these neurons were active (activation = 1) during phase A, and silent (activation = 0) during phase B. The remaining 80 neurons showed the reverse behavior. The network output layer size was 4, corresponding to the possible actions to navigate the maze (North, East, South, West). The learning rule and network architecture allowed us to study the spatial representations that emerge during learning the ABA renewal paradigm.

The network was trained to predict *Q*-values using the error backpropagation algorithm on sample experiences. The Adam optimizer was used during training to control the update speed, with a learning rate parameter of 10^−4^. Experience replay was used during training by sampling experiences consisting of (*s, a, r, s*′) tuples randomly from a replay buffer, if not stated otherwise. Experience replay is a mechanism commonly used in reinforcement learning to stabilize and improve the replay behavior, where experiences are randomly sampled from the history of previously seen state-action-reward experiences during training. In the condition with replay, we used a replay buffer large enough to store all previous experiences. In the condition without experience replay, experiences were randomly sampled from the last five trials for every training epoch.

The ε-greedy strategy was used to drive exploration during training, i.e., in every time step, the action corresponding to the highest *Q*-value was chosen with probability 1−ϵ and a random action with probability ϵ. ϵ was chosen to be 0.3 in all experiments.

### 2.4 Behavioral analysis

**Figure 2:**
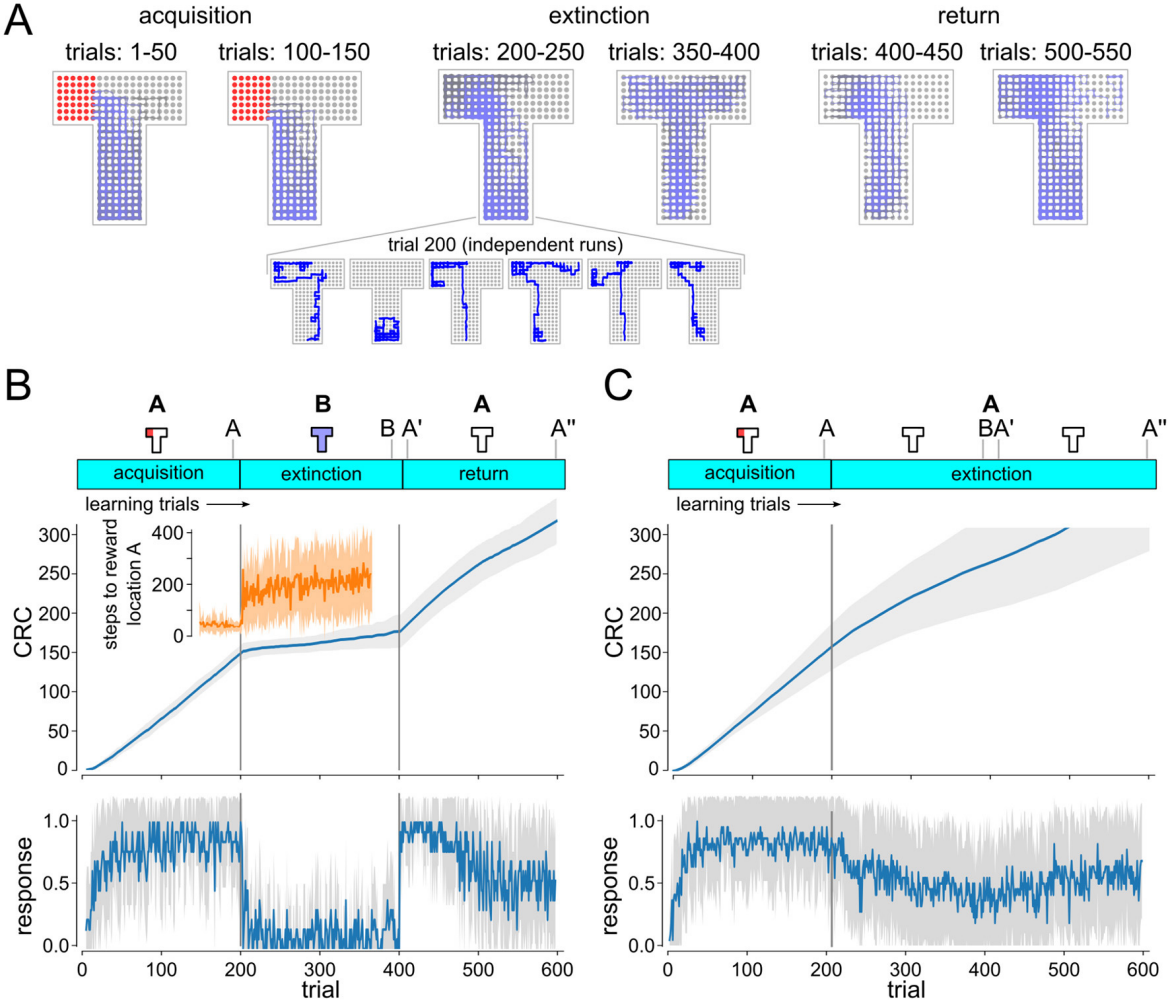
Analysis of the behavior of the simulated DQN agent. **(A)** Superposition of movement trajectories at different stages of the experiment. Colors indicate relative trial index (gray: earlier, blue: later). Reward locations indicated in red. Extinction and renewal in simulated agent. Inset shows individual extinction trajectories. **(B, C)** Cumulative response curves (CRC) ABA renewal **(B)** and extinction experiment **(C)**. Plots on the bottom show mean responses (increments of CRCs). Shaded area indicates STD over independent runs. Progression of extinction and renewal tasks and population vector recording time points (A, B, A′, A″) are indicated on top. Renewal of conditioned behavior is clearly visible after return to A in ABA task **(B)** but not in AA task **(C)**. Inset shows time to context A reward location (mean and STD).

Behavioral trajectories (sequence of grid points visited in the maze) were recorded in all experiments. Figure 2A shows the superposition of 50 successive movement trajectories. Every trial was classified based on these trajectories as conditioned response (CR) trial or non-CR trials. For CR analysis, agent locations were tracked over single trials. Trials were counted as CR trials if the agent entered the left arm of the maze within the first 50 time steps. Learning dynamics were visualized using cumulative response curves (CRC) (Donoso et al., 2021). CRC plots in Figure 2 show averages over 20 independent experimental runs.

### 2.5 Place field analysis

To analyze emergent spatial representations (see Figures 1F–H for an illustration) we adopted the method from Vijayabaskaran and Cheng (2022). First, activity vectors were recorded from all neurons of hidden layers in the network by placing the agent at all possible grid point locations and heading directions. Cells that produced zero output under all condition were classified as silent cells. Cells that produced non-zero activity at some locations for at least one, but not all, heading directions were classified as partially active neurons. Context cells were identified as neurons that generated the same output value *a*_A_ for all locations in context A and for all heading directions, within a tolerance of 0.05% with respect to the maximum neuron activation, and similarly in context B *a*_B_, but *a*_A_≠*a*_B_.

Preferred firing centers were further analyzed for the remaining neurons. Activity vectors were smoothed with a Gaussian smoothing filter before a cluster analysis was performed for each heading direction. Neurons with activity that revealed more than two clusters or neurons that were active at more than 50% of the grid point locations were excluded from the analysis. Heading-direction specific tuning centers were then computed as the weighted average of grid point locations in the dominating activity cluster. Cells, for which the heading-direction specific tuning center exceeded the distance of four times the distance between grid points, were excluded. All neurons that were excluded in the above tests were classified as heading-direction modulated cells. The remaining neurons were classified as place-cell-like.

**Figure 3:**
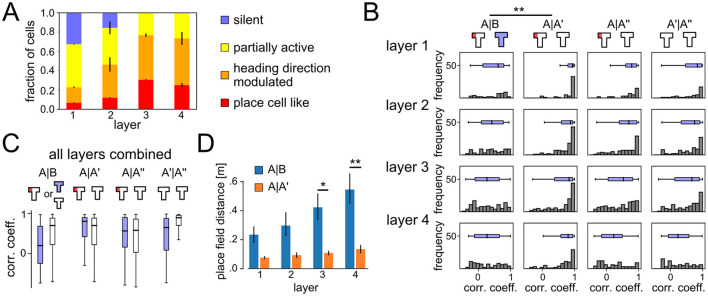
Emergent spatial representations and remapping. **(A)** Distribution of different cell types that emerged in the different network layers at the end of learning phase in context A. Deeper layers developed spatial coding more frequently. **(B)** Histograms of correlation coefficients between population vectors recorded at different phases of the ABA renewal paradigm. All layers show signs of global remapping, i.e., spatial correlations are low between contexts A and B, but high between two different exposures to the same context A. **(C)** Comparison of PV correlation coefficients for the ABA renewal paradigm (blue) and AA extinction (white). Data from all network layers are combined. Without the context change, spatial representations remain stable throughout extinction. **(D)** Distance between place cell centers for place-cell-like cells (mean ± SEM). *P*-values for two-sieded *T*-test < 0.1 (^*^) and < 0.05 (^**^).

For the remapping analysis in Figure 3, only neurons that were classified as place-cell-like in at least one of the experimental conditions were examined. Population vectors (PVs) were generated by concatenating the activity vectors of these neurons, merging the per-grid-point activities for the four heading directions into one. PVs were pooled together from 20 independent runs. Correlation coefficients were then computed on these PV amplitudes.

## 3 Results

### 3.1 Extinction and renewal dynamics

Walther et al. (2021) demonstrated that extinction and renewal can be reproduced in an *in-silico* model, using a simplified abstract ABA extinction learning paradigm. We first studied whether extinction and renewal behavior could be reproduced in a more complex simulated agent that interacts with a 3D environment. To do so, we designed a T-maze paradigm with a fixed reward location as the basis for the ABA renewal experiment (Figure 2). The agent is first placed in environment A (T-maze with reward zone in the left arm, white light) for 200 trials, before the behavior is extinguished in context B (T-maze without reward, blue light). We recorded behavioral trajectories and examined signs of extinction and renewal for the simulated agent in this ABA renewal paradigm.

Extinction and renewal emerged in the simulated agent when exposed to the ABA task (Figures 2A, B). Superposition of movement trajectories at different stages of the experiment shows that the agent quickly adopts the conditioned response (CR), i.e., prefers the left arm of the maze, entering the reward zone typically within the first 50 time steps of the trial (Figure 2A). After 200 trials, the agent was moved to context B (blue light) and reward was suspended. CR preference briefly continues after switch to B but quickly washes out to baseline, showing no preference between left and right arm (see Figure 2B). Response times were longer after transition to context B, which resulted in a sharp decline in conditioned response (Donoso et al., 2021), but the left preference persisted (see insets in Figures 2B, C). After returning to context A at trial 400 the CR is renewed followed by a slow gradual washout of CR. As in experiments, renewal requires the switch of context, i.e., if acquisition, extinction and test occur in the same context, there is no renewal (Figure 2C).

### 3.2 Emergent remapping of spatial representations

We next analyzed the emerging neural codes in the DQN agent after it has learned this task. The neural code analysis was performed at the end of every experimental phase. Based on these recordings and using a method adapted from Vijayabaskaran and Cheng (2022) (see Section 2.5), neurons of the DQN agent were classified into one of the four classes: silent, partially active, heading direction modulated, and place cell like. In addition, we identified context-dependent cells as neurons that generated different outputs in the two contexts, but showed no spatial tuning. Figure 1H shows example place fields extracted from the DQN. While place-cell-like responses could be found in all layers and experimental phases (Figure 3A), deeper layers had a larger number of spatially tuned neurons, e.g. in layer 3 30.62 ± 0.9% of the units were classified as place cell like, 46.24 ± 3.4% as heading direction modulated, and 23.12 ± 3.6% as partially active. A significant number of silent cells were found in layer 1 (32.58 ± 0.6%) and layer 2 (15.63 ± 13.2%) and these layers were overall less tuned to spatial features. Cells that responded exclusively to the context, without being also tuned to spatial features or heading direction, were only found in layer 1 and 2.

A new spatial code emerged when the agent was moved to context B as indicated by histograms of correlation coefficients for population vectors (PVs) from different experimental phases and network layers (Figure 3B). PVs in contexts A and B were only weakly correlated (histograms are flat or skewed toward 0), whereas correlations between PVs in context A at different time points (A, A′, A″) showed strong correlations with each other. Overall, deeper layers showed slightly stronger signs of remapping.

Figure 3C shows a comparison of correlation coefficients between the ABA and AA experiment. Remapping occurred in the ABA condition, whereas correlation coefficients were better explained by temporal proximity in the AA condition, suggesting a slow drift of neural representations rather than remapping. Our results suggest that global remapping in the spatial representations is a key mechanism underlying renewal.

The representations that emerge in the behaving agent are more compatible with the integrated code model than with a segmented code. The majority of neurons were activated in both contexts and modulated by spatial features or heading direction, consistent with a mixed representation. Only a small subset of neurons remained silent in either context A or B, or showed no spatial tuning. Interestingly, we found neurons that exclusively encoded context only in the first layers, close to the input. In particular, we examined neurons that produced the same output regardless of heading direction and the location of the agent. We found that 33.3± 0.78% of the neurons in layer 1 showed context cell behavior. Included in this count are 9.23 ± 0.28% (mean ± STD over 20 runs) of layer 1 neurons that were classified as silent cells in one context, but had non-zero, non-spatially tuned activity in the other, and 24.11 ± 0.73% of layer 1 neurons that were active in both contexts and had non-spatially tuned, context-dependent outputs. The number of context cells decreased rapidly with the layer number. In all 20 runs, only a total of four context cells were found in layer 2. Context cells and silent cells were absent in higher layers in all experimental conditions. This result shows that integrated codes better explain the neural representations that emerge in the behaving agent.

Of the neurons that showed place-cell-like behavior, only around 6% did so in both contexts A and B. The remaining ca. 94% were classified as silent cells, partially active, or heading direction modulated, in one of the contexts. To further study the remapping behavior, we measured the Euclidean distance between place field centers of cells that were active in both contexts (Figure 3D). There was a trend toward larger place field distances when comparing contexts A vs. B than for A vs. A′ across all layers, and the difference reached significance in the higher layers 3 and 4. This observation is compatible with global remapping in hippocampal recordings (Leutgeb et al., 2005).

### 3.3 The role of the behavioral task

Next, we analyzed the role of the behavioral task on the learning and remapping behavior. To do so, we repeated the above experiment with a similar ABA task acquisition setup. However, in this experiment we consider a relearning paradigm, thus during the second phase in context B, the right arm of the T-maze was rewarded and in the third phase, in context A, the reward location was returned to the left arm. If context is represented through integrated codes, different representations, that encode both task relevant spatial and contextual cues should emerge in training phases A and B.

**Figure 4:**
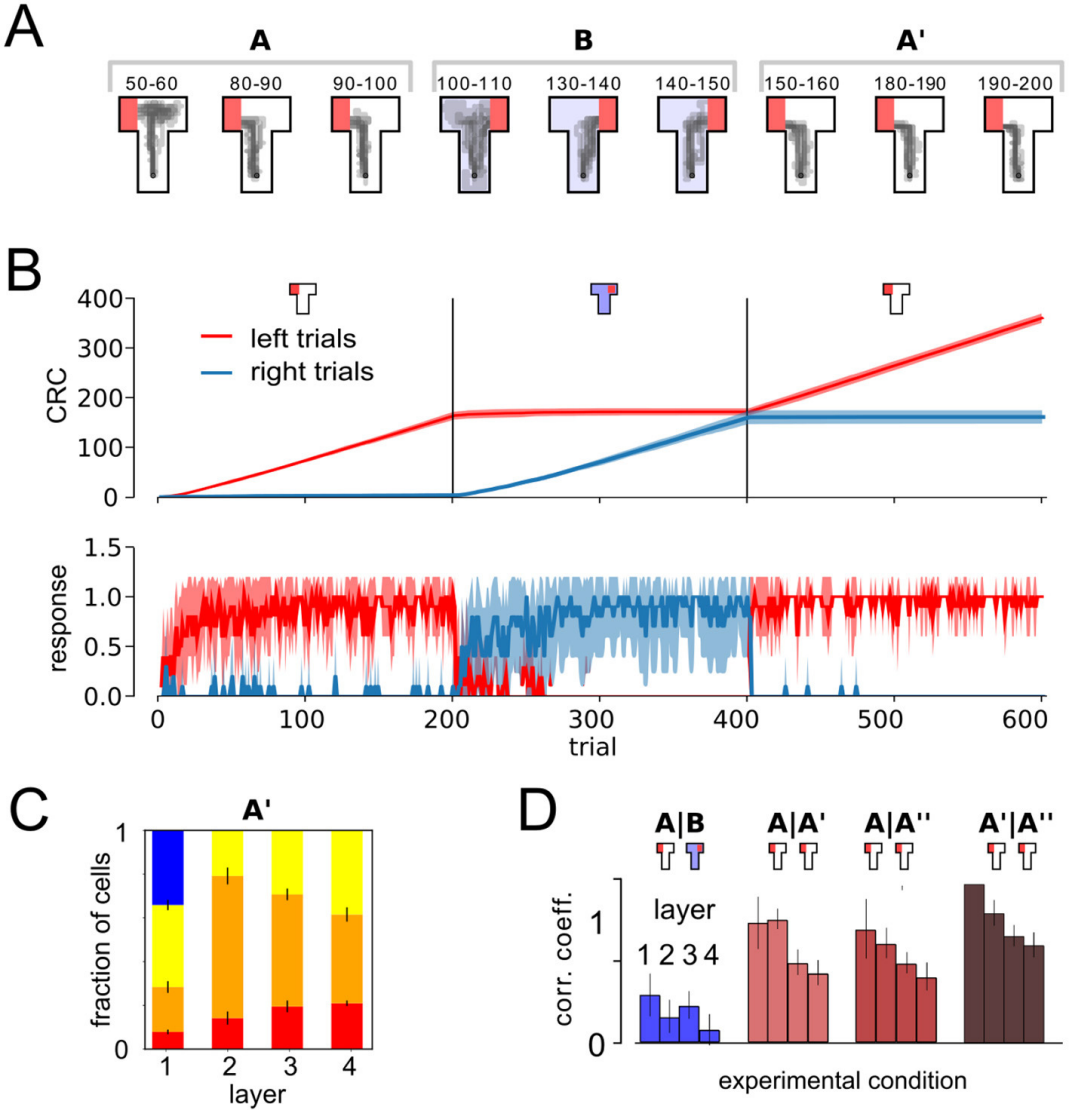
Remapping in a relearning paradigm. **(A)** Behavioral trajectories. **(B)** Cumulative response curves as in Figure 2 for the acquisition phase. The agent quickly acquired the rewarded response and immediately switched after return to context A (third phase). **(C)** Relative fraction of cell types as in Figure 3A. **(D)** Mean PC correlation coefficient per layer.

As in the previous setup, the agent was able to acquire the task within a few trials (Figure 4A). After rewarded maze arms are switched in phase B, right trials were reliably preferred (Figure 4B). Left preference is recovered immediately when the agent is restored to context A in the third phase. As in the previous experiment (Figure 3A), a significant number of cells, of around 20% in deeper layers, are classified as place-cell-like in this experiment (Figure 4C). As in the extinction task, population vectors in A and A′ phases are significantly stronger correlated than that of A and B, despite the long temporal distance, showing signs of place cell remapping (Figure 4D).

Although, context was crucial to the agent's behavior here, we again found an absence of context cells in higher layers of the DNN. In the relearning experiment, ~35% of the neurons in layer 1 qualified as context cells. Similar to the results above, 11.25 ± 0.37% (mean ± STD over 20 runs) of layer 1 neurons were silent in one context, buthad non-zero, non-spatially tuned activity in the other. 23.93 ± 0.64% of layer 1 neurons were active, and had non-spatially tuned, context-dependent outputs. On the other hand, only a small fraction of layer 2 and no layer 3 and 4 neurons showed context cell behavior, as in the extinction scenario above. This result shows that integrated codes emerge in the behaving agent, independent of the behavioral task.

### 3.4 The role of context salience in remapping and renewal

Our results in the preceding section suggest that remapping of task-relevant representations in the network is driving renewal in the test phase. To further investigate this dependency, we adapted the experiment to include a variable strength of context salience. The difference between the A and B contexts is determined by a change of illumination in the T-maze. To test the sensitivity of the emergent spatial representations to changes in the differentiability between A and B context, we repeated the experiment with different lighting conditions that blend between the A (white light) and B (blue light) context, parametrized by a new task variable (saliency of context, *s*). While context A remains fixed (white light), *s* = 0 indicates that both contexts are identical (white light in B), *s* = 1 corresponds to pure blue light in B, and values between 0 and 1 denote a mix of blue and withe light in context B. Clearly, *s* = 0 corresponds to extinction without context switch (Figure 2C).

**Figure 5:**
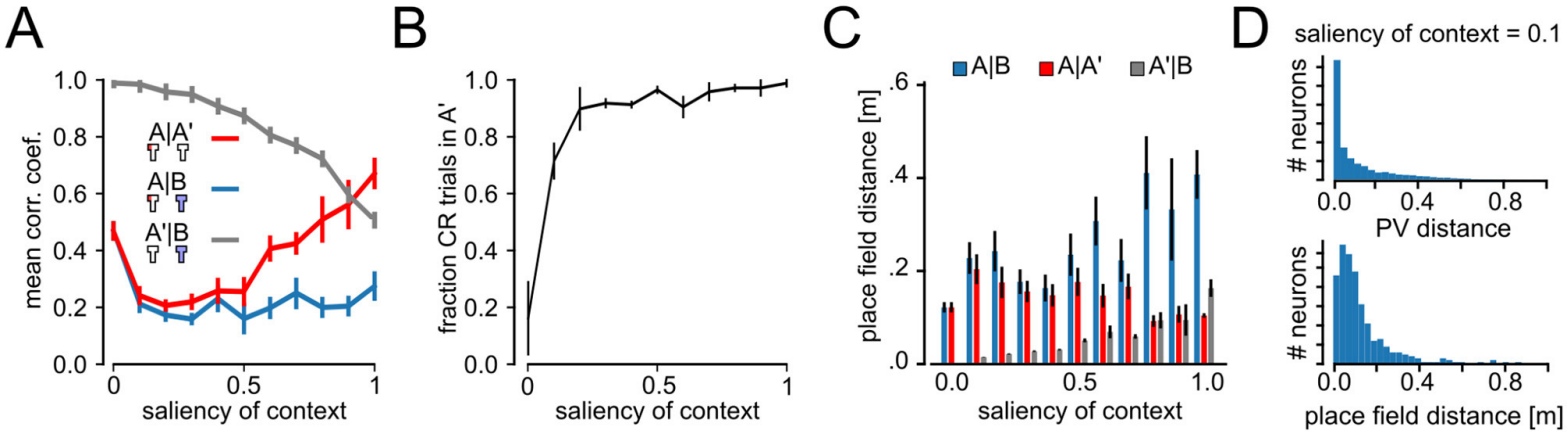
Impact of context salience on remapping and behavior. **(A)** Correlation coefficient between population vectors (PVs) taken at different phases of the experiment as a function of the context salience. A vs. A′ PVs show a U-shaped profile, suggesting interference between PVs for low and intermediate context salience. Correlation coefficient between A′ and B, to measure the interference between emerging neural patterns as a function of context salience. **(B)** Fraction of CR trials in the A′ phase (error bars show STD). **(C)** Distance between place cell centers for place-cell-like cells accross all layers for different context saliencies (mean ± SEM). **(D)** Histograms of A′–B PV distances and distance between place cell centers for context salience of 0.1.

We repeated the analysis of remapping in neural codes for experiments with different values of *s* (0 ≤ *s* ≤ 1) and compared mean correlation coefficients between PVs from experimental phases A, B, and A′ (Figure 5A). As expected correlations of PVs are identical between A–B and A–A′ for *s* = 0 (A, A′, and B conditions are indistinguishable) and maximally different for *s* = 1, suggesting high levels of remapping between context A and B. Interestingly, we found that A–A′ correlation coefficients showed a U-shaped profile. For *s* = 0, the correlations are high because the contexts A and B are identical and so are their representations. In that situation, extinction learning overwrites the acquired behavior so that there is no renewal. If context salience is low to intermediate (0 ≤ *s* ≤ 0.4), training in B causes interference with the previously learned spatial representations of A. For *s*>0.4, the two contexts are less similar and the interference between the spatial representations ameliorates. Despite the interference between spatial representations at low to intermediate context salience, renewal of CR could be observed already at quite low levels of context salience (Figure 5B). In Figure 5A we show the correlation coefficient between A′ and B, which is a measure of interference between the neural patterns needed to learn the behavior. As can be seen, interference also declines much slower than the rise in CR trials in Figure 5B.

In Figure 5C we analyzed the Euclidean distance between place field centers of cells, that were active in both contexts (as in Figure 3D) as a function of context saliency. As for PV correlations, place field distances for the A vs. B condition, were largest for runs with high saliency. Also A′ vs. B distances showed a similar behavior to correlation coefficients increasing steadily with the saliency of context. To further examine the mechanisms that enable renewal for low context saliency (Figure 3B), we studied the histograms of absolute PV distances and place field distance for A′–B with saliency of context 0.1. Most neurons show distances close to zero, while a few neurons had large distances of around 0.8 (Figure 5D). These findings suggest that at low context salience, spatial representations interfere, preventing a formation of clear segregated codes. However, differences in codes driven by a small subsets of neurons are strong enough to observe renewal also for low context saliency. For higher context salience (*s*>0.4), the agent learns distinct representations of A and B, leading to much higher A–A′ than A–B correlations, i.e., global remapping, and renewal.

### 3.5 Testing the role of an explicit context representation

**Figure 6:**
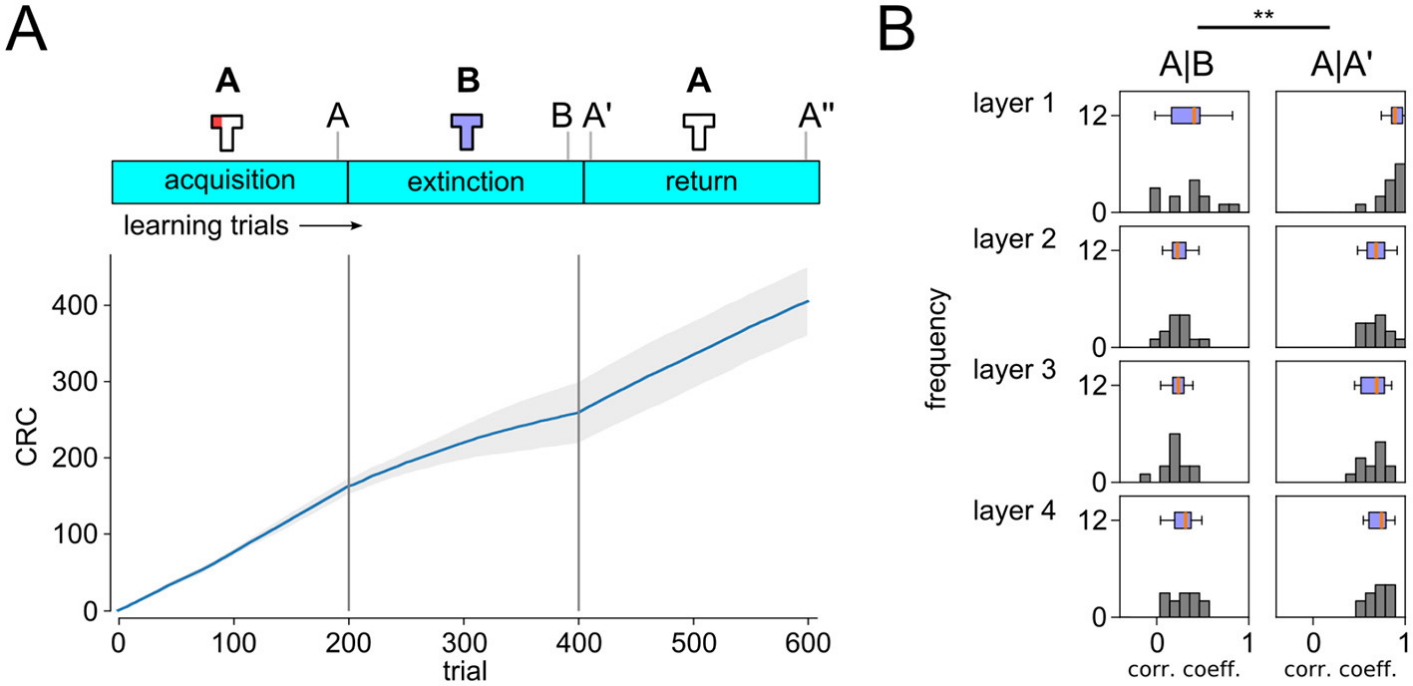
Extinction learning with segregated explicit context representation in the input. **(A)** CRC for ABA renewal experiment as in **(B)**. **(B)** Remapping analysis as in Figure 3B. Two-sided *T*-test *p*-values < 0.05 in all conditions (^**^).

To test the role of an explicit representation of context on the network behavior and neural codes, we repeated the extinction experiment with a modified input encoding, where context was presented through a distinct signal instead of changing the lighting in the camera inputs (see Section 2 for details). This modified agent was able to reliably learn the behavioral task and showed pronounced signs of extinction and renewal (Figure 6A). Importantly, we found an abundance of cells that encoded mixed representations, partially active, heading direction modulated and place-cell-like cells. As in our previous experiment, these cell types represented 100% of the neurons in layers 3 and 4. No cells were found that represented context only, despite this information being explicitly present in the input. Furthermore, as reported for the lighting context above, all layers showed significant signs of remapping (Figure 6B). This result shows that integrated codes emerge for different context representations even if context is included explicitly in the input, suggesting that integrated codes are the more likely outcome for a variety of behavioral learning tasks.

### 3.6 The role of experience replay in remapping

We next wondered what caused remapping in the DQN agent's neural representations. A simple hypothesis to explain the observed remapping behavior is that experience replay, that spans multiple experimental phases, rescues neural representations from forgetting and thus facilitates the emergence of two parallel spatial maps. This hypothesis would suggest that remapping would be abolished if replay were absent during training. Experience replay is a method commonly used in reinforcement learning to stabilize and improve learning (Ashraf et al., 2021). We use a very simple replay model that randomly samples experiences from the past during learning.

**Figure 7:**
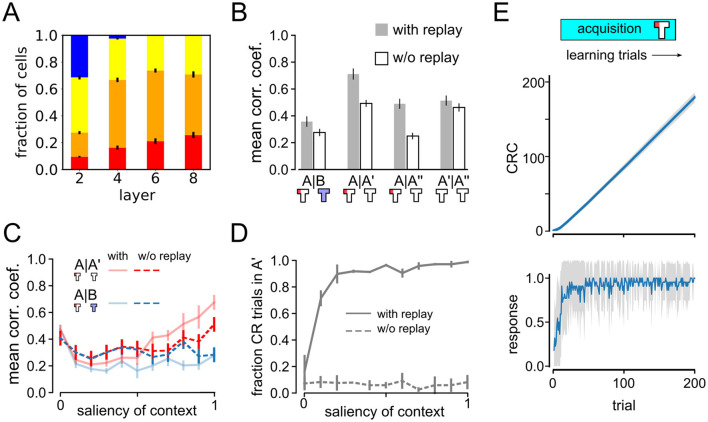
Impact of experience replay on remapping and behavior. **(A)** Relative fraction of cell types as in Figure 3A, but for an agent trained without experience replay. **(B)** Remapping analysis similar to Figure 3C showing less clear remapping when replay is absent. **(C, D)** Analysis of impact of context salience on remapping and behavior as in Figure 5 but for an agent trained without experience replay. Data without experience replay displayed with dashed lines. Solid lines show data with replay as in Figure 5 for comparison. **(E)** Cumulative response curves as in Figure 2 for the acquisition phase. The agent without replay was able to quickly acquire the rewarded response.

To test this prediction, we repeated the ABA renewal experiment with maximum context salience (*s* = 1), using a DQN agent without experience replay. Emergence of place cells did not depend on replay, as spatially tuned neurons also emerged in agents trained without experience replay (Figure 7A). Previous studies had already reported that place-cell like activity, among other neural dynamics, that resemble brain activity emerged in artificial autonomous agents navigating complex environments (Wyss et al., 2006). Vijayabaskaran and Cheng (2022) further studied the conditions required for place cell formation. Without replay PV correlations are overall decreased in all experimental phases, suggesting that experience replay has a stabilizing effect on neural representations (Figure 7B). A closer inspection of the relative correlation coefficients reveals that they are higher for A–A′ than for A–B, despite the fact that the latter are more closely spaced in time, indicating that global remapping occurs even in the no-replay condition. However, since the representations of A and B are less distinct from one another than in the simulations with replay, we conclude that replay helps in the formation of distinct representations during extinction learning in context B.

This conclusion is supported by another observation when comparing the representations of A, A′, and A″ (Figure 7B). There are roughly the same number of learning trials between A–A′ and A′–A″, but between A and A′ extinction occurs in context B whereas all trials between A′ and A″ occur in context A. Interestingly, without replay the A–A′ correlations are similar to A′–A″ correlations, suggesting that the context of the intervening trials has no influence on the stability of the representation of context A. However, if there is replay, A–A′ correlations are higher than *A*′–A″, where the latter is similar to the no-replay condition. This might seem counterintuitive, since between A and A′ extinction occur in a different context. To explain this observation, we note that the trials in the third experimental phase are not reinforced, so extinction learning should occur in context A. It is reasonable to assume that extinction learning leads to changes in neural representations to drive a different behavior. Our results suggest that if extinction learning occurs in a different context from acquisition, replay strengthens the representation of the acquisition context.

Finally, we studied the dependence between remapping and renewal in the no-replay condition by varying the context salience *s* (Figures 7C, D). Agents trained with experience replay (solid lines) are compared to those without replay (dashed lines). Without replay, remapping is apparent only for the highest context salience and not for lower values. On the other hand, no signs of renewal were found in the agents' behavior (Figure 7D). Importantly, the agent without replay was still able to acquire the rewarded behavior during the acquisition phase (Figure 7E). In summary, our modeling results suggest a crucial role of experience replay in renewal and an important role in remapping.

## 4 Discussion

In this study, we have shown that DQN agents trained on spatial tasks learn complex representations that encode both spatial position and contextual variables about the environment. We used an ABA renewal paradigm where a CR is extinguished in context B. We reproduced renewal after the agent is returned to context A across a wide range of experimental settings. We showed that complex context-dependent spatial representations and dynamics arise spontaneously in the hidden layer activity of a deep network when a spatial navigation task is learned. We found that the distinctness of contextual cues plays an important role in global remapping of spatial representations, and that the occurrence of global remapping strongly correlates with renewal behavior. Finally, we found that experience replay is critical to stabilize the acquired behavior during the extinction phase. Disabling experience replay also prevented renewal and reduced remapping.

These results demonstrate that deep-learning agents can reproduce a set of complex learning dynamics similar to those found in behaving animals. These results may therefore provide new insights to better understand how spatial representations are formed in the brain to support goal-directed behavior.

### 4.1 Plausibility of our modeling assumptions

Reinforcement learning provides a robust simulation framework for understanding the principles underlying navigation. As in biological systems, an RL agent has to cope with a number of complex problems such as interpreting sensory stimuli, extracting task-relevant features from these stimuli, or dealing with the exploration-exploitation trade-off. However, since reinforcement learning is driven by the goal of reward acquisition, it neglects other intrinsic learning mechanisms, such as curiosity or fatigue.

Furthermore, the combination of deep neural networks and reinforcement learning, which forms the basis of a DQN agent, makes it possible to process naturalistic inputs. However, the deep neural network in our model does not attempt to match the visual system of rodents and, therefore, it is difficult to compare the saliency of a visual change between our model and rodents. Hence, the degree of visual change that is required to drive global vs. rate remapping can also not be compared. However, for our purposes, it suffices that there are environmental changes that lead to global remapping in both animals and model agents. Furthermore, the DQN setup models neural activity only in an abstract way by generating analog neural outputs at each time step. These outputs model the activations of biological neurons at a coarse temporal resolution, roughly corresponding to neuronal firing rates or calcium transients. It will be interesting to study whether similar learning dynamics can be observed in more detailed models of biological neurons, such as Frémaux et al. (2013).

In order to maintain full control over the experimental setup, we studied a simple feed-forward DQN agent here. This choice was made because the aim of this study was not to model detailed temporal dynamics of neural activity, e.g. time cells and ramping cells (Lin et al., 2023). However, including recurrent connections in our model to study more complex temporal neural dynamics is straightforward and an interesting topic for future studies. Furthermore, we focused on a relatively abstract interaction with the environment, where only 4 different actions could be generated in every time step. By augmenting the model with more behavioral details, a number of additional experimental findings could be tested in future work. For example, experimental evidence indicates that hippocampal cognitive maps encode abstract task-relevant dimensions that encode value (Knudsen and Wallis, 2021), suggesting a complex mixed encoding of spatial representations, context, and task-relevant variables.

Despite its simplicity, the proposed model shares some features with the hierarchical processing of complex visual stimuli in the brain. Lower layers in the neural network are tuned to visual features, as expected in higher visual areas, whereas higher layers (neural layers close to the output) are tuned more to abstract representations that are invariant to detailed visual features or heading direction and show place-cell-like activity, in this sense resembling hippocampal areas. Furthermore, we used a very simple model of experience replay, and found it to be crucial for renewal. Experience replay is found in hippocampus during awake resting has been proposed as a mechanism for consolidating learnt experiences (Foster and Wilson, 2006; Buhry et al., 2011), and it plays a similar role in our simple reinforcement learning setup. However, we would like to emphasize that the aim of this study was not to match anatomical structures, or precise statistics of replay (Diekmann and Cheng, 2023) in detail, but to identify a minimum set of assumptions that would allow an agent to express the observed behavior and neural dynamics at a conceptual level.

As for the behavior, we focused on a T-maze paradigm, where the agent learns to navigate to a fixed local target to retrieve a reward. This experimental paradigm fits well with the reinforcement learning setup, and the DQN agent was able to quickly acquire this behavior. However, experimental studies have also explored other conditions for reliably inducing global or partial remapping, such as different maze topologies (Leutgeb et al., 2005; O'Keefe and Conway, 1978), odors (Wood et al., 1999), item-place associations (Komorowski et al., 2009), or sounds (Aronov et al., 2017). In future work, we will investigate whether remapping in DQN agents also occurs in scenarios that mimic these more diverse setups.

### 4.2 Spatial and contextual representations in the model network

In our model network, spatially tuned neurons were present more prominently in higher layers of the neural network, whereas lower layers were more strongly modulated by visual features, suggesting a hierarchical organization of neural codes. Signs of global remapping were found in all layers of the network. While replay had a strong effect on remapping and CR renewal, we found no significant difference in absolute number of spatially tuned cell types.

An interesting difference between lower and higher layers arises during extinction learning. As mentioned above, spatial representations change during extinction learning and replay helps to maintain the representation of the acquisition context during extinction in context B (see discussion of the results in Figure 7B). Since in the third experimental phase, trials are not reinforced to test for renewal in context A, the CR is eventually extinguished. To support this change in the behavior in context A, the neural representations have to change, too. Intriguingly, the change does not occur in the lower layers, where A–A″ correlations remain high (Figure 3B), but they do occur in the higher layers, where A–A″ are peaked around 0. This suggests that the sensory layers retain a stable representation of A, whereas the layers closer to the output have to change their representation to support a different behavior.

In our model, the occurrence of global remapping in the network is strongly correlated with renewal, nevertheless, for low values of the context salience, renewal occurs in the model even though our analysis of remapping does not indicate distinct representations of the two contexts (Figure 5B). We believe this is an artifact of using the correlations between the populations vectors in the two contexts (A–A′ and A–B) as an indicator of distinct representations. A similar phenomenon has been observed in other studies of memory storage and retrieval, where the similarity between patterns, as measured by a high correlation, did not sufficiently predict whether they would be confused in memory (Neher et al., 2015; Bayati et al., 2018).

While here we used a simplified feed-forward neural network to learn representations, a more detailed model that incorporates high-resolution temporal dynamics will be necessary to account for sub-second neural dynamics and complex neuron responses in the hippocampus in the future, e.g. the fast remapping dynamics on the temporal resolution of theta cycles (Jezek et al., 2011) and experience replay (Gillespie et al., 2021).

### 4.3 Testable experimental predictions

The proposed model makes a number of testable experimental predictions. First, our results suggest a close correspondence between remapping and renewal. This prediction could be tested in experiments where neural activity and behavior are recorded simultaneously. For instance, in a study with human participants, some individuals exhibit renewal, while other do not, and the occurrence of renewal correlated with differences in hippocampal activity (Lissek et al., 2013). This study together with our modeling results predict that in a spatial ABA renewal task, animals who exhibit renewal also show global remapping in the hippocampus, whereas those individuals who did not show renewal have stable spatial representations.

Second, we found that the salience of contextual cues plays an important role in extinction and renewal. It has already been shown in experiments that the property of remapping depends on the specificity of the change in experimental conditions (Latuske et al., 2018; Leutgeb et al., 2005). Our results predict a specific “U”-shaped dependence for the correlation coefficients between population vectors, i.e., it first declines and then rises again (see Figure 5A). It would be interesting to conduct an ABA renewal experiments with rats in a virtual reality environment that allows parametric changes to the environment. We predict that renewal correlates with the occurrence of global remapping in place cells.

Third, we find an important role for replay in the expression of renewal, which is consistent with several models that have previously explored the role of replay for learning (Johnson and Redish, 2005), exploration (Samsonovich and Ascoli, 2005), planning (Mattar and Daw, 2018), and active inference (Stoianov et al., 2022). Our finding suggests that disruption of replay (Gridchyn et al., 2020) would reliably attenuate renewal. If replay were disrupted in a context-specific manner, it would be possible to impair renewal in context A without affecting extinction learning in context B.

## 5 Conclusion

In summary, we have demonstrated the emergence of context-dependent spatial codes. These emerging representations in our model are compatible with the integrated model and with neural codes in the hippocampus. The occurrence of remapping is strongly correlated with renewal. This suggests that integrated codes are the mechanism for context-dependent extinction and renewal in the DQN agent.

## Data Availability

Publicly available datasets were analyzed in this study. This data can be found at: https://github.com/sencheng/CoBeL-RL.

## References

[B1] Aronov D. Nevers R. Tank D. W. (2017). Mapping of a non-spatial dimension by the hippocampal-entorhinal circuit. Nature 543, 719–722. 10.1038/nature21692 28358077 PMC5492514

[B2] Ashraf N. M. Mostafa R. R. Sakr R. H. Rashad M. Z. (2021). “A State-of-the-Art Review of deep reinforcement learning techniques for real-time strategy games,” in Applications of Artificial Intelligence in Business, Education and Healthcare, Studies in Computational Intelligence, eds. A. Hamdan, A. E. Hassanien, R. Khamis, B. Alareeni, A. Razzaque, and B. Awwad (Cham: Springer International Publishing), 285–307. 10.1007/978-3-030-72080-3_17

[B3] Auchter A. M. Shumake J. Gonzalez-Lima F. Monfils M. H. (2017). Preventing the return of fear using reconsolidation updating and methylene blue is differentially dependent on extinction learning. Sci. Rep. 7:46071. 10.1038/srep46071 28397861 PMC5387397

[B4] Bayati M. Neher T. Melchior J. Diba K. Wiskott L. Cheng S.. (2018). Storage fidelity for sequence memory in the hippocampal circuit. PLoS ONE 13:e0204685. 10.1371/journal.pone.0204685 30286147 PMC6171846

[B5] Bernier B. E. Lacagnina A. F. Ayoub A. Shue F. Zemelman B. V. Krasne F. B.. (2017). Dentate gyrus contributes to retrieval as well as encoding: evidence from context fear conditioning, recall, and extinction. J. Neurosci. 37, 6359–6371. 10.1523/JNEUROSCI.3029-16.2017 28546308 PMC5490069

[B6] Bouton M. E. (2004). Context and behavioral processes in extinction. Learn. Mem. 11, 485–494. 10.1101/lm.78804 15466298

[B7] Bouton M. E. Maren S. McNally G. P. (2021). Behavioral and neurobiological mechanisms of pavlovian and instrumental extinction learning. Physiol. Rev. 101, 611–681. 10.1152/physrev.00016.2020 32970967 PMC8428921

[B8] Buhry L. Azizi A. H. Cheng S. (2011). Reactivation, replay, and preplay: how it might all fit together. Neural Plast. 2011, 1–11. 10.1155/2011/203462 21918724 PMC3171894

[B9] Cochran A. L. Cisler J. M. (2019). A flexible and generalizable model of online latent-state learning. PLoS Comput. Biol. 15:e1007331. 10.1371/journal.pcbi.1007331 31525176 PMC6762208

[B10] Corcoran K. A. (2004). Factors regulating the effects of hippocampal inactivation on renewal of conditional fear after extinction. Learn. Mem. 11, 598–603. 10.1101/lm.78704 15466314 PMC523078

[B11] Diekmann N. Cheng S. (2023). A model of hippocampal replay driven by experience and environmental structure facilitates spatial learning. Elife 12:e82301. 10.7554/eLife.82301.sa2 36916899 PMC10076035

[B12] Diekmann N. Vijayabaskaran S. Zeng X. Kappel D. Menezes M. C. Cheng S.. (2023). CoBeL-RL: a neuroscience-oriented simulation framework for complex behavior and learning. Front. Neuroinform. 17:1134405. 10.3389/fninf.2023.1134405 36970657 PMC10033763

[B13] Donoso J. R. Packheiser J. Pusch R. Lederer Z. Walther T. Uengoer M.. (2021). Emergence of complex dynamics of choice due to repeated exposures to extinction learning. Anim. Cogn. 24, 1279–1297. 10.1007/s10071-021-01521-4 33978856 PMC8492564

[B14] Dunsmoor J. Niv Y. Daw N. Phelps E. (2015). Rethinking extinction. Neuron 88, 47–63. 10.1016/j.neuron.2015.09.028 26447572 PMC4598943

[B15] Foster D. J. Wilson M. A. (2006). Reverse replay of behavioural sequences in hippocampal place cells during the awake state. Nature 440, 680–683. 10.1038/nature04587 16474382

[B16] Frémaux N. Sprekeler H. Gerstner W. (2013). Reinforcement learning using a continuous time actor-critic framework with spiking neurons. PLoS Comput. Biol. 9:e1003024. 10.1371/journal.pcbi.1003024 23592970 PMC3623741

[B17] Fuhs M. Touretzky D. (2007). Context learning in the rodent hippocampus. Neural Comput. 19, 3173–3215. 10.1162/neco.2007.19.12.3173 17970649

[B18] Fujiwara H. Sawa K. Takahashi M. Lauwereyns J. Tsukada M. Aihara T.. (2012). Context and the renewal of conditioned taste aversion: the role of rat dorsal hippocampus examined by electrolytic lesion. Cogn. Neurodyn. 6, 399–407. 10.1007/s11571-012-9208-y 24082961 PMC3438325

[B19] George D. Rikhye R. V. Gothoskar N. Guntupalli J. S. Dedieu A. Lázaro-Gredilla M. (2021). Clone-structured graph representations enable flexible learning and vicarious evaluation of cognitive maps. Nat. Commun. 12:2392. 10.1038/s41467-021-22559-5 33888694 PMC8062558

[B20] Gershman S. J. Monfils M.-H. Norman K. A. Niv Y. (2017). The computational nature of memory modification. Elife 6:e23763+. 10.7554/eLife.23763 28294944 PMC5391211

[B21] Gillespie A. K. Astudillo Maya D. A. Denovellis E. L. Liu D. F. Kastner D. B. Coulter M. E.. (2021). Hippocampal replay reflects specific past experiences rather than a plan for subsequent choice. Neuron 109, 3149–3163.e6. 10.1016/j.neuron.2021.07.029 34450026 PMC8497431

[B22] Gridchyn I. Schoenenberger P. O'Neill J. Csicsvari J. (2020). Assembly-specific disruption of hippocampal replay leads to selective memory deficit. Neuron 106, 291–300. 10.1016/j.neuron.2020.01.021 32070475

[B23] Grieves R. M. Jeffery K. J. (2017). The representation of space in the brain. Behav. Processes 135, 113–131. 10.1016/j.beproc.2016.12.012 28034697

[B24] Hainmueller T. Bartos M. (2020). Dentate gyrus circuits for encoding, retrieval and discrimination of episodic memories. Nat. Rev. Neurosci. 21, 153–168. 10.1038/s41583-019-0260-z 32042144 PMC7115869

[B25] Heald J. B. Lengyel M. Wolpert D. M. (2021). Contextual inference underlies the learning of sensorimotor repertoires. Nature 600, 489–493. 10.1038/s41586-021-04129-3 34819674 PMC8809113

[B26] Jezek K. Henriksen E. J. Treves A. Moser E. I. Moser M.-B. (2011). Theta-paced flickering between place-cell maps in the hippocampus. Nature 478, 246–249. 10.1038/nature10439 21964339

[B27] Ji J. Maren S. (2008). Differential roles for hippocampal areas CA1 and CA3 in the contextual encoding and retrieval of extinguished fear. Learn. Mem. 15, 244–251. 10.1101/lm.794808 18391185 PMC2327266

[B28] Johnson A. Redish A. D. (2005). Hippocampal replay contributes to within session learning in a temporal difference reinforcement learning model. Neural Netw. 18, 1163–1171. 10.1016/j.neunet.2005.08.009 16198539

[B29] Jones B. W. Deem J. Younts T. J. Weisenhaus M. Sanford C. A. Slack M. C.. (2016). Targeted deletion of AKAP7 in dentate granule cells impairs spatial discrimination. Elife 5:e20695. 10.7554/eLife.20695 27911261 PMC5135391

[B30] Knudsen E. B. Wallis J. D. (2021). Hippocampal neurons construct a map of an abstract value space. Cell 184, 4640–4650.e10. 10.1016/j.cell.2021.07.010 34348112 PMC8459666

[B31] Komorowski R. W. Manns J. R. Eichenbaum H. (2009). Robust conjunctive item-place coding by hippocampal neurons parallels learning what happens where. J. Neurosci. 29, 9918–9929. 10.1523/JNEUROSCI.1378-09.2009 19657042 PMC2746931

[B32] Kubie J. L. Levy E. R. J. Fenton A. A. (2020). Is hippocampal remapping the physiological basis for context? Hippocampus 30, 851–864. 10.1002/hipo.23160 31571314 PMC7954664

[B33] Latuske P. Kornienko O. Kohler L. Allen K. (2018). Hippocampal remapping and its entorhinal origin. Front. Behav. Neurosci. 11:253. 10.3389/fnbeh.2017.00253 29354038 PMC5758554

[B34] Leutgeb S. Leutgeb J. K. Barnes C. A. Moser E. I. McNaughton B. L. Moser M.-B.. (2005). Independent codes for spatial and episodic memory in hippocampal neuronal ensembles. Science 309, 619–623. 10.1126/science.1114037 16040709

[B35] Lin D. Huang A. Z. Richards B. A. (2023). Temporal encoding in deep reinforcement learning agents. Sci. Rep. 13:22335. 10.1038/s41598-023-49847-y 38102369 PMC10724179

[B36] Lissek S. Glaubitz B. Uengoer M. Tegenthoff M. (2013). Hippocampal activation during extinction learning predicts occurrence of the renewal effect in extinction recall. Neuroimage 81, 131–143. 10.1016/j.neuroimage.2013.05.025 23684875

[B37] Mattar M. G. Daw N. D. (2018). Prioritized memory access explains planning and hippocampal replay. Nat. Neurosci. 21, 1609–1617. 10.1038/s41593-018-0232-z 30349103 PMC6203620

[B38] McNaughton B. L. Barnes C. A. Gerrard J. L. Gothard K. Jung M. W. Knierim J. J.. (1996). Deciphering the hippocampal polyglot: the hippocampus as a path integration system. J. Exp. Biol. 199(Pt 1), 173–185. 10.1242/jeb.199.1.173 8576689

[B39] Neher T. Cheng S. Wiskott L. (2015). Memory storage fidelity in the hippocampal circuit: the role of subregions and input statistics. PLoS Comput. Biol. 11:e1004250. 10.1371/journal.pcbi.1004250 25954996 PMC4425359

[B40] O'Keefe J. Conway D. H. (1978). Hippocampal place units in the freely moving rat: why they fire where they fire. Exp. Brain Res. 31, 573–590. 10.1007/BF00239813 658182

[B41] Pavlov I. P. (1927). Conditioned Reflexes: An Investigation of the Physiological Activity of the Cerebral Cortex. New York, NY: Oxford University Press. 10.5214/ans.0972-7531.1017309 PMC4116985 25205891

[B42] Penny W. D. Zeidman P. Burgess N. (2013). Forward and backward inference in spatial cognition. PLoS Comput. Biol. 9:e1003383. 10.1371/journal.pcbi.1003383 24348230 PMC3861045

[B43] Peters J. Dieppa-Perea L. M. Melendez L. M. Quirk G. J. (2010). Induction of fear extinction with hippocampal-infralimbic BDNF. Science 328, 1288–1290. 10.1126/science.1186909 20522777 PMC3570764

[B44] Plitt M. H. Giocomo L. M. (2021). Experience-dependent contextual codes in the hippocampus. Nat. Neurosci. 24, 705–714. 10.1038/s41593-021-00816-6 33753945 PMC8893323

[B45] Redish A. D. Elga A. N. Touretzky D. S. (1996). A coupled attractor model of the rodent head direction system. Network: Comput. Neural Syst. 7:671. 10.1088/0954-898X/7/4/00415673682

[B46] Redish A. D. Jensen S. Johnson A. Kurth-Nelson Z. (2007). Reconciling reinforcement learning models with behavioral extinction and renewal: implications for addiction, relapse, and problem gambling. Psychol. Rev. 114, 784–805. 10.1037/0033-295X.114.3.784 17638506

[B47] Rescorla R. A. (1972). “A theory of pavlovian conditioning: Variations in the effectiveness of reinforcement and non-reinforcement,” in Classical Conditioning, Current Research and Theory, Vol. 2, eds. A. H. Black, and W. F. Prokasy (New York, NY: Appleton- Century-Crofts), 64–69.

[B48] Rosas-Vidal L. E. Do-Monte F. H. Sotres-Bayon F. Quirk G. J. (2014). Hippocampal-prefrontal BDNF and memory for fear extinction. Neuropsychopharmacology 39, 2161–2169. 10.1038/npp.2014.64 24625752 PMC4104333

[B49] Samsonovich A. McNaughton B. L. (1997). Path integration and cognitive mapping in a continuous attractor neural network model. J. Neurosci. 17, 5900–5920. 10.1523/JNEUROSCI.17-15-05900.1997 9221787 PMC6573219

[B50] Samsonovich A. V. Ascoli G. A. (2005). A simple neural network model of the hippocampus suggesting its pathfinding role in episodic memory retrieval. Learn. Mem. 12, 193–208. 10.1101/lm.85205 15774943 PMC1074338

[B51] Sanders H. Wilson M. A. Gershman S. J. (2020). Hippocampal remapping as hidden state inference. eLife 9:e51140. 10.7554/eLife.51140 32515352 PMC7282808

[B52] Soliman F. Glatt C. E. Bath K. G. Levita L. Jones R. M. Pattwell S. S.. (2010). A genetic variant BDNF polymorphism alters extinction learning in both mouse and human. Science 327, 863–866. 10.1126/science.1181886 20075215 PMC2829261

[B53] Stoianov I. Maisto D. Pezzulo G. (2022). The hippocampal formation as a hierarchical generative model supporting generative replay and continual learning. Prog. Neurobiol. 217:102329. 10.1016/j.pneurobio.2022.102329 35870678

[B54] Taniguchi A. Fukawa A. Yamakawa H. (2022). Hippocampal formation-inspired probabilistic generative model. Neural Netw. 151, 317–335. 10.1016/j.neunet.2022.04.001 35468492

[B55] Vijayabaskaran S. Cheng S. (2022). Navigation task and action space drive the emergence of egocentric and allocentric spatial representations. PLoS Comput. Biol. 18:e1010320. 10.1371/journal.pcbi.1010320 36315587 PMC9648855

[B56] Walther T. Diekmann N. Vijayabaskaran S. Donoso J. R. Manahan-Vaughan D. Wiskott L.. (2021). Context-dependent extinction learning emerging from raw sensory inputs: a reinforcement learning approach. Sci. Rep. 11:2713. 10.1038/s41598-021-81157-z 33526840 PMC7851139

[B57] Wang Q. Wang Q. Song X.-L. Jiang Q. Wu Y.-J. Li Y.. (2018). Fear extinction requires ASIC1a-dependent regulation of hippocampal-prefrontal correlates. Sci. Adv. 4:eaau3075. 10.1126/sciadv.aau3075 30417090 PMC6223961

[B58] Whittington J. C. Muller T. H. Mark S. Chen G. Barry C. Burgess N.. (2020). The tolman-eichenbaum machine: unifying space and relational memory through generalization in the hippocampal formation. Cell 183, 1249–1263. 10.1016/j.cell.2020.10.024 33181068 PMC7707106

[B59] Wood E. R. Dudchenko P. A. Eichenbaum H. (1999). The global record of memory in hippocampal neuronal activity. Nature 397, 613–616. 10.1038/17605 10050854

[B60] Wyss R. König P. Verschure P. F. J. (2006). A model of the ventral visual system based on temporal stability and local memory. PLoS Biol. 4:e120. 10.1371/journal.pbio.0040120 16605306 PMC1436026

[B61] Zelikowsky M. Hast T. A. Bennett R. Z. Merjanian M. Nocera N. A. Ponnusamy R.. (2013). Cholinergic blockade frees fear extinction from its contextual dependency. Biol. Psychiatry 73, 345–352. 10.1016/j.biopsych.2012.08.006 22981655 PMC3525775

[B62] Zhang K. (1996). Representation of spatial orientation by the intrinsic dynamics of the head-direction cell ensemble: a theory. J. Neurosci. 16, 2112–2126. 10.1523/JNEUROSCI.16-06-02112.1996 8604055 PMC6578512

